# Timing precision of the Individual Differences in Dutch Language Skills (IDLaS-NL) test battery

**DOI:** 10.3389/fnhum.2025.1625756

**Published:** 2025-09-17

**Authors:** Janay Monen, Olha Shkaravska, Peter Withers, Johan Weustink, Maarten van den Heuvel, Paul Trilsbeek, Reiner Dirksmeyer, Antje S. Meyer, Florian Hintz

**Affiliations:** ^1^Psychology of Language Department, Max Planck Institute for Psycholinguistics, Nijmegen, Netherlands; ^2^Donders Institute for Brain, Cognition and Behaviour, Radboud University, Nijmegen, Netherlands; ^3^Research Center Deutscher Sprachatlas, Marburg University, Marburg, Germany; ^4^Center for Mind, Brain and Behavior, Marburg University & Justus Liebig University Giessen, Giessen, Germany

**Keywords:** online testing, individual differences, test battery, timing precision, Language Skills

## Abstract

Online experimentation has become an essential tool in cognitive psychology, offering access to diverse participant samples. However, remote testing introduces variability in stimulus presentation and response timing due to differences in participant hardware, browsers, and internet conditions. To ensure the validity of online studies, it is crucial to assess the timing precision of experimental software. The present study evaluates the Individual Differences in Dutch Language Skills (IDLaS-NL) test battery, a collection of online tests designed to measure linguistic experience, domain-general cognitive skills, and linguistic processing. Implemented using Frinex, a programming environment developed at the Max Planck Institute for Psycholinguistics, IDLaS-NL allows researchers to customize test selections via a web platform. We conducted two studies to assess the timing precision of five chronometric tests within the battery. In Study 1, we evaluated the initial implementation of the tests, analyzing differences between expected and recorded stimulus presentation times, response latencies, and recording delays using the custom-made Web Experiment Analyzer (WEA). The results indicated imprecisions in some measures, particularly for reaction time and audio recording onset. Visual stimulus presentation, on the other hand, was fairly accurate. Study 2 introduced refined timing mechanisms in Frinex, incorporating specialized triggers for stimulus presentation and response registration. These adjustments improved timing precision, especially for speech production tasks. Overall, our findings confirm that Frinex achieves timing precision comparable to other widely used experimental platforms. While some variability in stimulus presentation and response timing is inherent to online testing, the results provide researchers with useful estimates of expected precision levels when using Frinex. This study contributes to the growing body of research on online testing methodologies by offering empirical insights into timing accuracy in web-based experiments.

## Introduction

Online experimentation has become a vital part of behavioral research in psychology, including psycholinguistics. Compared to the lab, running studies remotely via the internet greatly facilitates data collection (e.g., Fairs and Strijkers, 2021; Garcia et al., 2022; Pinet et al., 2017; Reips, 2000). The potential of online testing is particularly evident in individual differences research, which requires assessing large and diverse samples of participants. Online experimentation differs from in-lab testing in that participants are not instructed and supervised by trained experimenters and in that their equipment is often inferior to that of dedicated psychological laboratories. As a result, data collected online tend to be noisier than data collected in the lab (see Crump et al., 2013; Germine et al., 2012; McConnell et al., 2025; Al-Salom and Miller, 2019; Semmelmann and Weigelt, 2017; Rodd, 2024). While detailed task instructions may mitigate the lack of an experimenter in remote test settings (Rodd, 2024), measurement noise (i.e., variability in the timing of stimulus presentation and in the registration of participant responses) due to properties of the participants’ computers or their browsers remains a challenge (Garaizar et al., 2014; Bridges et al., 2020; Anwyl-Irvine et al., 2021). To distinguish variability stemming from differences in participants’ abilities or skills from hardware- and software-related noise, researchers need to know to what extent stimulus and response events are affected by timing imprecision. Timing precision refers to how consistently a web platform records responses or presents stimuli at pre-set intervals. To illustrate, imagine a visual attention task with a stimulus X, which is programmed to appear for a duration of precisely 400 ms across trials. In order to achieve good timing precision, the actual duration of stimulus X would have to stay consistently close to those 400 ms for every single trial. However, if, for instance, the duration of stimulus X shows 250 ms for some trials and 550 ms for others, this would introduce noise into the reaction time data and thus point to a low timing precision.

The present paper concerns the timing precision in the Individual Differences in Dutch Language Skill (IDLaS-NL) test battery. This is a collection of tests designed to capture variability in linguistic experience and language processing and in domain-general skills implicated in using language (Hintz et al., 2025). The tests are implemented as online tests, using Frinex, a software package developed at the Max Planck Institute fur Psycholinguistics. They are freely available to the research community. Researchers can create customized versions of the battery by selecting subsets of the tests on a dedicated web platform^1^ (Hintz et al., 2024). Here, we focus on the precision of stimulus timing and the measurement of response latencies achieved with Frinex. We describe two studies in which we assessed slightly different implementations of the tests. The versions described in Study 1 concern the tests as run in Hintz et al. (2025); the versions described in Study 2 concern the tests as implemented on the web platform, available to the research community (Hintz et al., 2024).

The IDLaS test battery was developed for adult speakers of Dutch. Based on the hypothesis that variability in linguistic processing is influenced by variability in linguistic experience and general cognitive skills (McQueen and Meyer, 2019), the battery comprises chronometric and accuracy-based tests to assess word and sentence comprehension, linguistic experience (which leads to linguistic knowledge), processing speed, working memory, and non-verbal reasoning. With the exception of non-verbal reasoning, each construct is assessed in several tests. We administered the battery to a validation sample of 748 participants (Hintz et al., 2025). A total of 169 of these participants completed the tests in a laboratory at the Max Planck Institute for Psycholinguistics using hardware optimized for experimentation, and the remaining 579 participants completed the same tests at home on their own laptops via the internet. Confirmatory factor analyses suggested good construct validity for all psychological constructs. In addition, our analyses provided evidence for measurement invariance for all but the processing speed constructs^2^ when comparing in-lab and remote participants. That is, although the absolute values for the chronometric tests differed (e.g., response times were generally longer in the online than the lab-tested sample), the factor loadings and model diagnostics did not differ significantly between them (see also McConnell et al., 2025).

While our measurement invariance tests provide important evidence for the suitability of the battery for online use, they do not provide estimates of the extent to which the chronometric test scores were influenced by noise resulting from online test administration. This issue has been investigated for other platforms (e.g., Anwyl-Irvine et al., 2021; Bridges et al., 2020; Garaizar et al., 2014), but not for the platform used for the IDLaS-NL battery. We therefore conducted two studies that assessed the timing precision for five chronometric tests in this battery. These tests involve combinations of trial events that are representative of all tests in the battery. The first study was run alongside data collection for the validation sample (Hintz et al., 2025). Based on the results of this study, we adjusted the implementation of the experiments to further improve timing precision and ran a second study. The results from Study 1 reported below show to what extent timing imprecision contributed measurement noise to the data reported in Hintz et al. (2025). The results from Study 2 provide users of our web platform with estimates of the extent to which chronometric test performance is subject to timing imprecision.

### Brief introduction to the Framework for interactive experiments (Frinex)

Frinex has been designed and developed since 2015 by Peter Withers at the Max Planck Institute for Psycholinguistics Nijmegen for online web experiments, and for offline field experiments since 2016 (Withers, 2022, 2024). It is under active development, and features can be added to meet novel requirements. Stimuli can be shown in Frinex as written words, texts, or pictures (still or animated) spoken words or texts and in video. All visual elements can be customized using cascading style sheets or using various predefined styles. Participant responses can be recorded in a number of ways, including button presses, typing, speaking or video-recording. Stimulus presentation and responses are time-stamped.

Each experiment in Frinex is built as a discreet application that does not change unless explicitly recompiled. Based on the researcher’s needs, an experiment can consist of multiple sub-applications, namely, a web, mobile, and a desktop version, as well as an admin application. The web application can be used via a web browser, and the mobile application can be deployed in various app stores and used on Android and iOS mobile devices. The alternative to the web application is the desktop application, which is created through Electron, an open-source software framework that uses JavaScript, HTML, and CSS to render a Chromium browser engine that can be run on both Windows and macOS. This allows participants to run the experiment on their local machine. Additionally, Electron controls for variation in browser performance, which is important considering the vast differences in browser performance that have been described (Anwyl-Irvine et al., 2021). In the present studies, we used Electron 10 with Chromium version 85.0.4183.121 (Frinex version 1.4.2917-stable).

Frinex itself consists of a number of components; of relevance are the build and deployment system, the experiment and its admin interface. Each experiment is defined using an Extensible Markup Language (XML) file. This XML file contains the visual layout and styling information, the logical flow of the experiment, and the stimulus selection. Any media files that are used, including stimulus files, are stored in a directory alongside the XML file.

Once the XML file contains all necessary elements, it is deployed. During this stage, the XML is validated and translated into source code (Java, JavaScript), which is compiled into independent applications for web, mobile and desktop, alongside the corresponding database storing experimental data. The build system is the driving force that automates the compilation and deployment processes. Important to note is that the deployment process is separated into discrete targets of staging and production. More specifically, the staging deployment of an experiment is only for institute-internal use when designing the experiment. When the experiment is run with participants, it is deployed to “production.”

Each compiled experiment has a dedicated database and admin interface. A network connection is required to transmit the collected data to the admin database. The transmission of data from the experiment to the admin interface is tolerant of network interruptions and will automatically upload data as it is connected. The data collected in the admin database can be accessed at any time including live, i.e., while an experiment is running. The data can be viewed in various ways: via a web browser directly in the admin interface, downloaded as a collection of CSV files, or queried dynamically in JSON format. Collected media recordings, audios and videos, can be viewed in the admin interface, downloaded individually or in ZIP files sorted by collection date. The recommended method of accessing the data is via the JSON REST interface, since this interface can be queried via R or NumPy, for example. The JSON REST interface is self-documenting.

In Frinex, timing is handled in two ways: The first and simplest one, which was implemented in Study 1 below, is by using the <pause> element, which will delay an upcoming trial event for at least the specified period (e.g., a <pause> of 500 ms before each trial is equal to a delay of 500 ms). Note that the delay resulting from using pause is approximate. Another way to time events, which was used in Study 2 below, is by choosing a time base as reference of the events, such as media playback time or audio recording time, for which a <addMediaTrigger> or <addFrameTimeTrigger> element can be used. Both of these elements will trigger when the media time passes the pre-set time interval (e.g., triggering the presentation of a trial item 500 ms after the recording has started). If the event is triggered later than the specified threshold value in milliseconds, an <onError> will be triggered.

## Study 1

### Methods

To assess priming precision of the IDLaS-NL tests in Frinex, we used a similar approach to that of Anwyl-Irvine et al. (2021). We selected five tests and ran them on two laptops, one featuring a Windows operating system (win10 × 64) and one featuring a Mac operating system (macOS 12.2), for more details see Table 1. Using two different laptops allowed us to assess how variability in hardware affected timing precision. The tests were chosen to include visual and auditory stimulus presentation, constant and variable stimulus timing, and spoken as well as manual responses.

**TABLE 1:** Technical details of the laptops used in Study 1 and 2.

Hardware type	OS	CPU	RAM	Hard drive
Windows (2016)	win10 × 64	Intel Core i5 4,200 m	4 GB	500 GB HDD
Apple MacBook Pro (2020)	macOS 12.2	2 GHz 4 core Intel	16 GB LPDDR4	512 GB M.2 SSD

The tests were run in an automated mode simulating human participants. To mimic a manual response, a button was triggered automatically after a specific time interval, and to mimic a spoken response a tone was played. We aimed for 50 datasets (i.e., simulated participants) per Windows laptop and 35 datasets per Mac laptop. From our experience with online experimentation, this distribution approximates the distribution of Windows and Mac laptops in the Dutch participant pool.

### Measurement hardware

**FIGURE 1:**
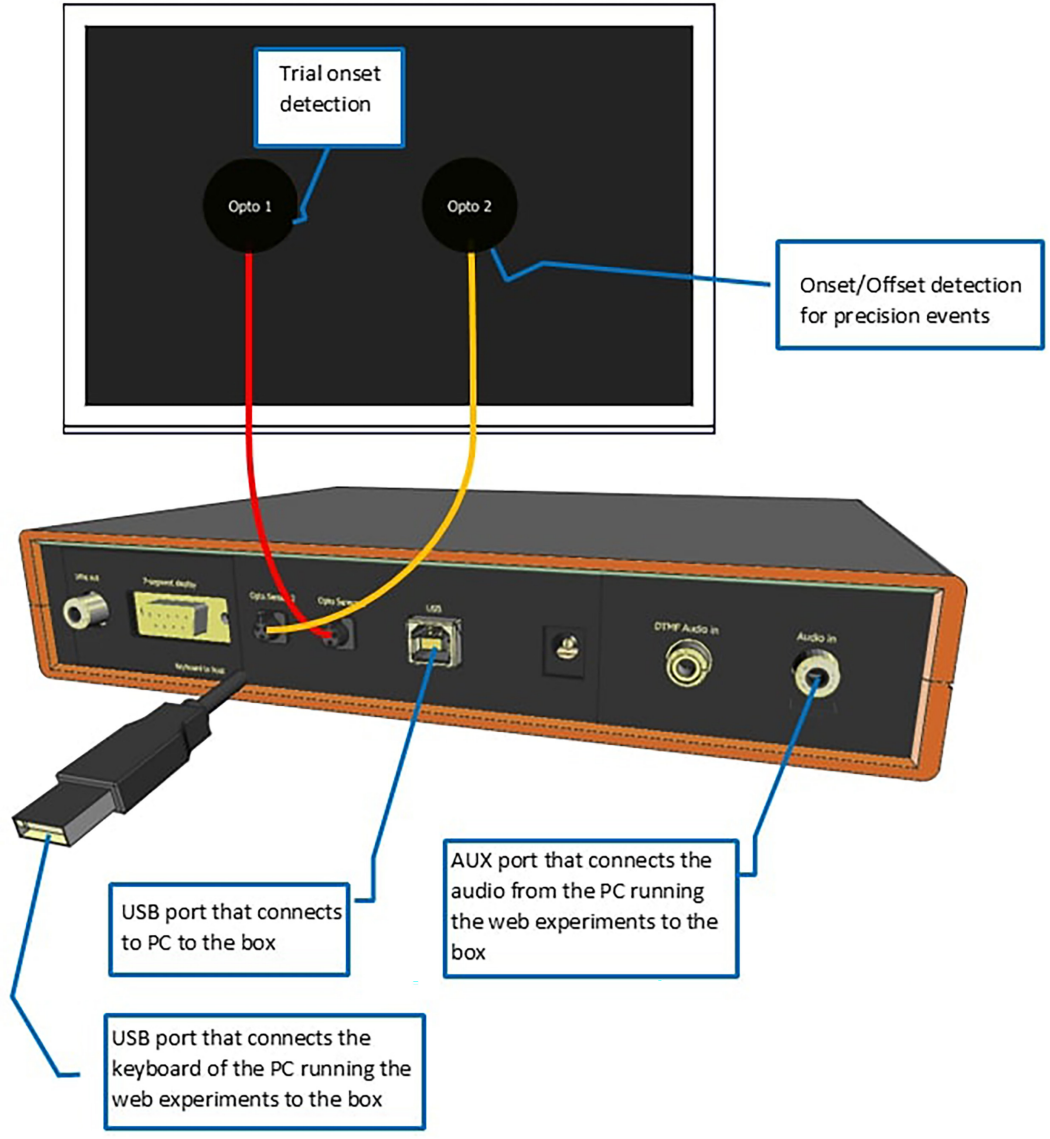
Schematic of the Web Experiment Analyzer. Please refer to Supplementary materials 1 for a construction manual of the WEA.

Each laptop was connected to the Internet via “eduroam” WIFI, hosted at the Max Planck Institute for Psycholinguistics. They were connected to the Web Experiment Analyzer (WEA, for short, see Figure 1). WEA is a box-shaped apparatus, which was custom-made at the Max Planck Institute for Psycholinguistics. It uses an Arduino Uno micro controller board and has a user interface that allows the user to measure the onsets and offsets of acoustic and visual stimuli, and the onsets of participant responses.

For the simulations, we created two visual stimuli, specifically a black fixation cross and a black square, both centered on the screen. They were shown with variable durations, as described below. WEA detected their onsets and offsets through two optical sensors (Opto1 and Opto2 hereafter) attached to the laptop screen. Opto1 detected the onset of each trial and served as a reference. The auditory stimulus was a.wav file of a 550 Mhz sine tone with a duration of 400 ms. Its onset was detected by Opto2, as soon as the Frinex application had started media playback. This feature was embedded in the experiment XML as element <mediaplaybackstarted>.

The participants’ responses were created using built-in software of the WEA. To simulate a spacebar press for the relevant experiments, a spacebar-click generator was used, which created clicks at different predefined times. WEA detected the click onset through Opto2.

To simulate speech, we used a beep generator, which created a dual-tone multi-frequency (DTMF) tone of 550 Mhz, with a duration of 400 ms. It was output through an external speaker, recorded using a Sennheiser microphone and stored as.wav file. This happened at a sampling rate of 44,100 Hz, 16-bit resolution. The volume was set to mimic speech as produced by the participants, at approximately 75 dB. For speech trials, we also measured the onset of the recording interval (which in the actual tests corresponded to the onset of the stimulus the participant responded to and hence co-determined the response latency). This was done by channeling the DTMF signal to the box and to the external loudspeaker. The box registered the signal while the web recorder, embedded in the experiment XML, recorded it via an external microphone.

### Test descriptions

To create trial timing in Study 1, we used the <pause> element in the XML configuration of every test described below. Here, we focus on the description of the stimulus and response types and the timing of the events in a trial. For detailed information about each test, please refer to Hintz et al. (2025). The trial timing for each test is shown in Supplementary Appendix A.

#### Auditory simple reaction time (ASRT)

The auditory simple reaction time task assesses the participant’s ability to respond quickly to the onset of auditory stimuli. At the start of each trial, a fixation cross is presented on the screen. After a variable interval (1,000–3,000 ms), a 550 Mhz sine tone with a duration of 400 ms is presented. The participant responds as quickly as possible by pressing a pre-defined response button. One second after the button-press, the next trial begins.

For the simulation, we ran 20 test trials per “participant.” Following the presentation of the tone, the robot participant triggered the spacebar after a predetermined interval of 220 ms, which corresponded to the average response latency of participants in a large pilot study (Hintz et al., 2020). WEA measured the precision of three events. The first was the *precision of visual stimulus duration* (the fixation cross duration). This event refers to the time interval between the onset of the fixation cross and the onset of the auditory stimulus, i.e., the sine tone (in ms). Note that the presentation duration for the fixation cross varied across trials, and a goal of this simulation was to assess how well Frinex would deal with this variability.

The second measure was the *precision of auditory stimulus presentation onset*, which is the difference between the expected onset of the sine tone and its actual onset (in ms), as recorded by WEA. The last measure was the *precision of reaction time*, which was set to be triggered at 220 ms after audio onset. For each measure, we expected values of 0 ms, which is equal to no delay.

#### Visual simple reaction time (VSRT)

The visual simple reaction time task assesses the participant’s ability to respond quickly to the onset of visual stimuli. As in the auditory version, a fixation cross is presented at the start of the trial. After a variable interval (between 1,000 and 3,000 ms) it is replaced by a line drawing of a square with white contours. The participant responds as quickly as possible by pressing a pre-defined response button. One second after the button-press the next trial begins.

For the simulation, we ran 20 test trials per “participant.” After the presentation of the visual stimulus, the spacebar was set to trigger at 240 ms to imitate a human response (Hintz et al., 2020).

We measured the precision of two trial events. *Precision of visual stimulus duration* was measured as the difference between the onset of the fixation cross and the onset of the white square. The target time interval between the transition from fixation cross to the visual stimulus varied randomly between 1,000 and 3,000 ms. The second measurement was *precision of reaction time*, calculated as the difference between the onset of the white square and the onset of the spacebar press (in ms). For each measure, we expected values of 0 ms, which is equal to no delay.

#### Picture naming

In the picture naming test, participants name line drawings of everyday objects as quickly as possible. A fixation drawing of a common object scaled to 300 × 300 pixels is presented for three seconds. The trial ends after a further 1,000 ms.

For the simulation, there were 40 test trials in total. As this test required the production of speech, a DTMF tone was included to the mimic speech onset. Once WEA detected a picture on the screen, the web-recorder started recording and the DTMF tone was played after 900 ms to simulate the spoken response of the artificial speaker.

We measured the precision of two events. The first one was the *duration of the fixation cross*, measure from the onset of the fixation cross to the onset of the line drawing. The second event was the *audio recording onset*, i.e., the moment when the web-recorder started recording. Here, WEA measured the difference between the onset of the DTMF tone and the onset of the recording (in ms). We expected values of 0 ms, which is equal to no recording delays. To obtain speech output, the DTMF tone (replacing the speech of the participants) was recorded and saved as.wav file at a sampling rate of 44,100 Hz, 16-bit resolution. The recording parameters were identical in all speech production tests in this study.

#### Structured sentence generation

The structured sentence generation test is a sentence production task that assesses participants’ ability to generate descriptions of scenes varying in structure and complexity. Each trial starts with a fixation cross in the center of the screen for 200 ms, followed by the presentation of a written verb (an html text label) presented for the duration of 500 ms. Immediately afterward, a fixation cross replaces the verb for 200 ms, followed by a colored image of two persons involved in an action. The participant describes the action using the written verb and presses the spacebar as soon as they have completed the sentence. After a one-second inter-trial interval, the next trial begins.

For the simulation, we generated 80 test trials. Participant responses were obtained as in the picture naming test described above. Speech onset was replaced by the onset of a DTMF tone, which played simultaneously with the onset of the line drawing (200 ms after fixation cross presentation), and manual responses were given by the robot 4,200 ms after target picture onset.

We measured the precision of two events: The first one was the *duration of the written verb*, measured from its onset to the onset of the colored image. The expected duration was 500 ms. The other event was the simulated speech onset, that is the onset of the recording (in ms) relative to the onset of DTMF. Values of 0 ms reflect exact timing precision in written verb presentation and recording onset.

#### Verbs semantics activation during sentence comprehension

This sentence comprehension test uses a simplified version of the classic visual word paradigm (Altmann and Kamide, 1999). Each trial starts with a fixation cross shown in the center of the screen for 800 ms, followed by the presentation of two common objects shown on the left and right side of the screen. A spoken sentence follows, which refers to one of the objects. The participant presses the left or right response button depending on the location of the mentioned object.

For the simulation, 40 trials were created. First, a fixation cross appeared in the center of the screen for 800 ms, after which the two objects were shown. This was followed by a pause with a random duration between 1,500 and 2,000 ms. After this pause, the pre-concatenated 550 Mhz tone started, representing the sentence onset. A response was simulated by triggering the spacebar 4,961 ms after Opto2 offset (i.e., sentence onset).

We measured the precision of four events. The first one was the *duration of the fixation cross*, measured from its onset to the onset of the display showing two images. This should be 800 ms. The second was *pause duration*, for which a specific duration between 1,500 and 2,000 ms was expected for each trial. Pause duration was the time interval between the onset of the two-object display and the tone, standing in for a spoken sentence. A third measure was the *precision of tone onset*, measured by comparing the expected to the actual audio onset, which was based on the variable pause duration per trial. This determined whether there was any audio delay. The final event measured was the *precision of reaction time*, i.e., the spacebar response. Again, we expected values of 0 ms for each trial event difference.

### Analysis

In sum, there were eight trial events for which we determined the timing precision: *visual stimulus duration*, *auditory stimulus presentation* (audio delay, cue delay and sentence delay), *audio recording onset*, *precision of pause duration* and *manual reaction time*. As mentioned before, WEA measurements for each of these events served as the standard, reflecting the intended timing. These values were compared to the timestamp values as recorded in the Frinex experiment database. The outcome of the timing precision events that we report in the next section are the result of subtracting the intended WEA timing measurements from the timing measurements as provided by Frinex.

There were two types of outliers. The first type were observations that deviated by more than 500 ms from the intended time or duration, which were eliminated from the analyses. For the second type, we followed Anwyl-Irvine et al. (2021) in that any value that was more than four times the standard deviation (SD) from the mean was excluded as well (see Tables 2, 3). After this step, new means and SD were calculated. This process was carried out for each variable. The resulting means and SD are provided for each device.

**TABLE 2:** Summary of the timing precision results of Study 1 for each of the five experiments, including mean, standard deviation (SD) and percentiles for each precision event.

Task	Measures		Percentiles			Mac		Windows	
	Mean	SD	25%	50%	75%	Mean	SD	Mean	SD
**1. Auditory simple reaction time**									
5 outliers removed	–	–	–	–	–	–	–	–	–
Fixation cross	−2	11	−10	−2	5	1	10	−6	10
Audio delay	31	17	20	29	40	28	21	34	11
Reaction time	113	17	104	111	117	115	20	111	9
**2. Visual simple reaction time**									
3 outliers removed	–	–	–	–	–	–	–	–	–
Fixation cross	−5	18	−19	−8	9	6	17	−17	8
Reaction time	81	8	76	81	86	83	8	80	6
**3. Picture naming**									
6 outliers removed	–	–	–	–	–	–	–	–	–
Fixation cross	3	13	−4	−3	13	12	12	−4	8
Record delay	−66	16	−78	−64	−53	−54	8	−78	11
**4. Structured sentence generation**									
6 outliers removed	–	–	–	–	–	–	–	–	–
Target verb	−5	12	−17	−3	2	−13	12	−0.02	8
Record delay	−85	11	−92	−85	−77	−82	11	−88	11
**5. Verb semantics activation during comprehension**									
22 outliers removed	–	–	–	–	–	–	–	–	–
Fixation cross	−4	12	−11	−6	0	4	14	−9	8
Pause	34	15	25	33	41	28	20	37	9
Audio delay	29	18	15	31	42	15	19	37	11
Reaction time	136	27	113	125	163	141	28	119	10

Values reflect the difference between Web Experiment Analyzer (WEA) and Frinex measurements in milliseconds.

**TABLE 3:** Timing precision (mean, SD, range) in milliseconds in Study 2.

Task	Measures		Percentiles			Mac		Windows	
	Mean	SD	25%	50%	75%	Mean	SD	Mean	SD
**1. Auditory simple reaction time**									
6 outlier removed	–	–	–	–	–	–	–	–	–
Fixation cross	6	16	−6	6	18	18	10	−6	11
Audio delay	25	19	10	24	39	33	22	31	12
Reaction time	113	17	104	110	117	116	21	110	9
**2. Visual simple reaction time**									
2 outliers removed	–	–	–	–	–	–	–	–	–
Fixation cross	7	14	−1	5	18	18	9	−4	8
Reaction time	84	7	81	82	88	84	9	83	4
**3. Picture naming**									
34 outliers removed	–	–	–	–	–	–	–	–	–
Fixation cross	7	11	−2	3	19	14	10	−2	1
Recording delay	59	13	48	64	71	47	8	71	5
**4. Structured sentence generation**									
149 outliers removed	–	–	–	–	–	–	–	–	–
Target verb	−3	13	−10	2	3	−8	19	2	6
Recording delay	80	13	76	83	86	69	11	87	10
**5. Verb semantics activation during sentence comprehension**									
450 outliers removed	–	–	–	–	–	–	–	–	–
Fixation cross	7	11	−1	3	19	17	10	0.07	3
Pause	24	72	15	29	37	10	130	32	8
Audio delay	26	20	12	31	40	21	23	14	9
Reaction time	116	17	106	114	121	118	23	114	10

## Results and discussion

Summary statistics (means and SD) for the five tests, combined across the two laptops and for each laptop individually are reported in Table 2. The results per laptop (MacBook vs. Windows) are also visualized in Supplementary materials 2. As Table 2 shows, across all experiments, precision was best, in terms of means and SD, for *visual stimulus duration*, ranging between mean differences of −5 and 3 ms. It was poorest for the *reaction time* measures (mean differences between 81 and 136 ms). Importantly, the SDs for the *reaction time* measure, ranging between 8 and 27 ms, suggest that these delays were fairly consistent. The results further showed that the *audio playback* started approximately 30 ms (SD = 17–18) later and that *pauses* between stimulus events lasted approximately 34 ms (*SD* = 15) later than intended. Interestingly, the analyses showed that *audio recordings* started between 85 and 66 ms (*SD* = 11–16) *earlier* than intended.

Turning to the individual devices, we see that in general, both show fairly similar patterns for the majority of the precision events, with only slight differences. In general, the SDs appear to be smaller on the Windows device. Note, however, that this could be a result of the larger number of participants simulated on this device. Furthermore, *reaction time* measures were more precise on the Windows laptop across all five tests, both in terms of mean and SD. The MacBook, on the other hand, had shorter *audio delays*, more precise *fixation cross presentation* and shorter delays for the *pause presentation*. The MacBook was also more consistent in starting *audio recordings*, albeit that – as for the Windows laptop – these started before the intended point in the trial.

That audio recordings were started *before* the intended point in the trial, had been observed for other programming platforms (e.g., jspsych, de Leeuw, 2015) as well. When working on audio recording features for jspsych, de Leeuw (2015), personal communication observed that the MediaRecorder API in a browser started to record about 100 ms of data before the recording was requested to start. We will return to this issue in Study 2.

In sum, the results of Study 1 suggest that timing precision in Frinex is similar to that of other programming platforms used for online experimentation (e.g., Gorilla). For example, Anwyl-Irvine et al. (2021) reported that the average delay for presenting visual stimuli ranged between −6 and 26 ms across four (partly commercial) platforms. Similarly, in their study, reaction time measures showed imprecisions, in the order of average delays ranging between 71 and 87 ms across platforms. The values we observed for these measures in the present study were very similar. In addition to these measures, we provide estimates for audio playback, audio recording and pause duration delays in Frinex.

## Study 2

To achieve event timing, the tests in Study 1 relied on the <pause> command, an element that creates a delay of a specified value, after which the next trial event occurs. For example, when implementing the time between fixation cross presentation and audio playback, we specified <pause = 300 ms>. This method relies on the browser’s ability to respond within the specified time when triggering trial events and has to do with the internal workings of the browser. More specifically, pauses are generic timers that can be applied to any event in the experiment and are not necessarily synchronized to each other, something which – with the wisdom of hindsight – could have led to inaccuracies in the imprecise timing of some trial events.

After the completion of Study 1, a set of new features were added to Frinex. These included the timers <addFrameTimeTrigger> and <addTimerTrigger> to create event timing and <addMediaTrigger> to create event timing when a media file is involved. Contrary to the <pause> elements, both of these types of timers are tied to the specific event for which timing precision should be achieved and are thus better synchronized to each other. We tested the impact of this change in Study 2.

## Materials and methods

The hardware setup was identical to that in Study 1. Per laptop, we simulated 50 participants. The same five experiments were evaluated. However, the internal structure was slightly adjusted. That is, we implemented the <addFrameTimeTrigger>, <addMediaTrigger>, and <addTimerTrigger> timers. <addFrameTimeTrigger> is a type of timer that is used to evaluate its triggers before each frame is rendered in the browser. The ASRT and VSRT, Structured sentence generation, and the Verb semantics activation during sentence comprehension tests used this timer. The <addMediaTrigger> timer adds a media recording/playback event that will trigger when the media first passes the provided milliseconds value. This timer was included in the Picture naming and Sentence generation test. The last timer, <addTimerTrigger>, was used to preserve a timing event even if the page needed to be refreshed. The ASRT test used this timer.

In contrast to the previously used generic <pause> timer, the new timers rely on animation frame and media frame callbacks. The animation frame callback synchronizes an event with the screen’s refresh rate and ensures that visual updates (e.g., presenting a stimulus) occur right before refreshing the screen, which allows for better timing precision. Media frame callback synchronizes events with media playback frames such as audio, which occurs when the timing of an action should coincide with a specific frame of a media file. This ensures a discrete time unit synchronized with media playback. In both of these cases, an accuracy threshold (20 ms is the minimum recommended value) must be provided and an error will be triggered if the event does not occur within that threshold. Thus, in general, trials should proceed more smoothly than with the previous timer. If such an error occurs, it is likely that other issues would occur on that browser or computer configuration.

Aside from the addition of the three timers, WEA was upgraded to help improve the accuracy of determining the audio onset for the Picture naming and Structured sentence generation tests: Study 1 required the generation of a 550 Mhz test tone of 400 ms to determine the audio onset for these two speech production tests. As a result of the upgrade to WEA, a tone was now automatically triggered by optical sensor Opto2 with the Beeper Action function of the box software. This tone was then directed to the microphone of the Windows laptop and to the audio input for the MacBook during Picture Naming. For the Structured sentence generation test, the tone was directed to the audio input for both laptops. Apart from these changes, the setup and the analysis of Study 2 were identical to those of Study 1.

## Results and discussion

The summary statistics for Study 2, averaged across both devices and for each device individually, are reported in Table 3. Supplementary materials 3 visualize the results for the two laptop types. Precision was best for *visual stimulus duration* in terms of mean and SD, ranging between mean differences of −3 and 7 ms. It was poorest for the *reaction time* measures (mean differences between 84 and 116 ms). As in Study 1, the SDs for the *reaction time* measure, ranging between 7 and 17 ms, suggest that these delays were fairly consistent. The *audio playback* in Study 2 started approximately 26 ms (SD = 20) later than intended. The *pause duration* had a very similar delay (24 ms), its SD, however, was substantially larger (SD = 72). The analyses further showed that *audio recordings* started on average between 59 and 80 ms (SD = 13) *later* than intended.

Turning to the individual devices, we see that for some measures, precision became more similar across the Windows and MacBook notebooks. This concerns the *reaction time* and *audio delay* measures. The reaction time measures ranged between 83 and 118 ms (ASRT: 116 vs. 110 ms, VSRT: 84 vs. 83 ms, Verb semantics: 118 vs. 114 ms). The audio delays ranged between 14 and 33 ms (Picture naming: 33 vs. 31 ms, Structured sentence generation: 21 vs. 14 ms). Less similar was the precision for *visual stimulus presentation* (18 vs. ∼−3 ms), with the Windows laptop outperforming the MacBook. *Pause duration* (10 vs. 32 ms) and *audio recording start* (47/69 vs. 71/87 ms), however, were more precise on the MacBook.

A comparison of the results of Study 1 and Study 2 shows that most improvements were found for the speech production experiments (i.e., the *audio recording start* measure), most likely a result of adding <addMediaTrigger>. While in Study 1 recordings started *before* the intended event in the trial, in Study 2, we only observed positive differences.

Another noteworthy result concerns the *visual duration* measure. Whereas in Study 1 we only observed one single positive average delay value (for picture naming), we only found positive delays for Study 2 (when considering averages across both devices). This, again, seems to imply that adding the <addFrameTimeTrigger> or the <addTimerTrigger> prevented the fixation cross from being presented for a shorter time than requested.

Similarly, slight improvements were found for the *precision of auditory stimulus presentation* and *precision of pause duration* events, both of which included either the <addFrameTimeTrigger> or the <addTimerTrigger>. The improvements for these measured events generally differed between 2 ms to 13 ms from the results of Study 1, which indicates that the changes were relatively small and may not necessarily have been caused by the implementation of new timers. Updates to the WEA could also explain these changes. However, this cannot be said with certainty.

Looking at the overall range of mean reaction time measurements, the values were very similar but slightly more precise (ranging between 84 and 116 ms deviance) than the measurements found for Study 1 (ranging between 81 and 136 ms deviance).

Finally, we note that the number of outliers excluded in Study 2, particularly in the Verb semantics activation during sentence comprehension task (450 outliers) and the Structured sentence generation task (149 outliers), was relatively high. Motivated by this pattern, we examined the distribution of outliers in Study 1 and 2 for each device and trial event. This overview can be found in Table 4. The excessive number of outliers in Study 2 for timing accuracy of the fixation cross in the Verb semantics activation during comprehension test relates to the Mac device. Importantly, the same device did not produce similar numbers of outliers for the same trial event (fixation cross) in other tests featuring optimized Frinex code (Study 2). For example, the number of outliers for the fixation cross presentation in the A/SRT tests for the Mac device in Study 2 was zero. Moreover, the same device produced only one outlier for the fixation cross presentation in the Verb semantics activation during comprehension test in Study 1. These numbers render it unlikely that general code compatibility issues are the origin of these outliers. Moreover, it also seems unlikely that the outliers are related to the updated code as – on this account – also other tests in Study 2 should be affected.

**TABLE 4:** Summary of timing precision outliers by Mac and Windows devices in Study 1 and Study 2 for each of the precision events across five experiments.

Task	Study 1			Study 2		
	Mac	Windows	Total	Mac	Windows	Total
**1. Auditory simple reaction time**						
Fixation cross	0	0	0	0	0	0
Audio delay	0	2	2	1	1	2
Reaction time	0	3	3	0	4	4
	–	–	**5**	–	–	**6**
**2. Visual simple reaction time**						
Fixation cross	0	1	1	0	1	1
Reaction time	0	2	2	0	1	1
	–	–	**3**	–	–	**2**
**3. Picture naming**						
Fixation cross	1	1	2	1	27	28
Record delay	0	4	4	2	4	6
	–	–	**6**	–	–	**34**
**4. Structured sentence generation**						
Target verb	5	0	5	41	54	95
Record delay	1	0	1	1	53	54
	–	–	**6**	–	–	**149**
**5. Verb semantics activation during comprehension**						
Fixation cross	1	3	4	357	46	403
Pause	1	7	8	29	6	35
Audio delay	2	3	5	1	6	7
Reaction time	0	5	5	0	5	5
	–	–	**22**	–	–	**450**

The values in bold font indicate the total number of outliers that were removed.

*Post hoc* investigations and reviewing the lab notebook, made it clear that the large number of outliers is due to a human error, specifically a sub-optimal placement of Opto2 on the Mac laptop screen. Due to this setup error, the markers did not change color as planned, resulting in more extreme values than one can expect if Opto2 had been in optimal position. It is important to highlight that (1) this was the only instance and that (2) the descriptive statistics based on the remaining data fit well with values recorded for the same trial event in other tests. Most importantly for future users of the platform, these outliers were not related to Frinex, but to an error in the experimental setup to measure its timing precision.

In spite of the unexpectedly large number of outliers, the overall improvements in timing precision observed across most measures in Study 2 confirm the positive effects of the new timers. With the wisdom of hindsight, the advantages of the new timers are obvious as the <pause> element only serves as a convent delay method and is not expected to provide precise timestamps for events. Our studies have shown that the <pause> element should be replaced with the new timers when timing is important since it does not use the animation and media frame callbacks provided by the browser. Both of which are designed to provide timestamps when handling animations and media files. With these changes implemented, Frinex represents a robust and promising platform for online psycholinguistic experimentation.

## Conclusion

The aim of this study was to assess the timing precision of different trial events as implemented tests of the Individual Differences in Dutch Language Skills test battery IDLaS-NL (Hintz et al., 2025, 2024). To that end, we selected a subset of the tests in the battery that feature auditory and visual stimulus presentation and spoken and manual responses and computed the timing precision using a custom-made device.

Consistent with earlier studies, we found that the *precision of visual stimulus duration* was the most consistent and resulted in the smallest delays, whereas the *precision of reaction time* contributed the most noise and was less constant across experiments. By incorporating more refined timer structures in Study 2, we improved timing precision for most of the measured precision events, with the use of <addMediaTrigger> resulting in the biggest improvement for those experiments that require speech production. Although the overall results were generally similar across the two operating systems, we observed some key differences in the precision of specific measurements, which reflect the inherent characteristics of each system. Windows (2016) demonstrated greater precision in reaction time measurements, with consistently smaller delays observed when compared to the MacBook (2020). This difference highlights the potential for variability in timing precision based on the operating system used, with Windows providing slightly more consistent results for tasks requiring fast response times, such as those testing reaction speed or decision-making. While this result aligns with observed variability across platforms in previous studies (e.g., Anwyl-Irvine et al., 2021), it is important to note that the discrepancies observed here were relatively small and unlikely to affect the overall outcomes of studies using Frinex.

In contrast, the MacBook (2020) showed superior performance in terms of record delay, with more accurate and consistent results in this particular measure. Specifically, the MacBook was more precise in initiating the recording of audio and visual stimuli, which is crucial for experiments involving speech production or the timing of media playback. This suggests that MacBook systems may offer advantages in tasks where accurate recording is essential, such as those involving vocal responses or other time-sensitive media events. These platform-specific strengths further highlight that Frinex offers flexible and reliable performance across a range of devices, making it suitable for a variety of experimental designs.

While the observed differences in reaction time precision and record delay may reflect the unique performance profiles of Windows and Mac devices, they do not significantly impact the overall timing precision or the reliability of Frinex. In fact, the robustness of the platform is evident in the fact that these differences are relatively minor and fall within the typical range of variability seen in other online experimental platforms (see, for instance, Bridges et al., 2020). Researchers should be mindful of these small discrepancies when planning studies that rely on precise timing, but they can be confident that Frinex offers reliable and accurate results regardless of the operating system used.

The precision for visual stimulus presentation reported here is comparable to values reported for other platforms (Garaizar et al., 2014; Bridges et al., 2020; Anwyl-Irvine et al., 2021). The precision for reaction times (generally above 100 ms) appears to be slightly lower, with values around 70 ms or 80 ms found for other platforms. Overall, however, we conclude that IDLaS-NL and Frinex, the programming environment used to deliver it, are well-suited for research purposes.

Future work could explore whether further software (in Frinex or other platforms) or hardware improvements might help minimize the observed discrepancies. For example, optimizing the code execution pathways or incorporating hardware-specific calibration routines could potentially reduce the reaction time variability observed, particularly on devices that currently exhibit less precise performance. In addition, integrating adaptive algorithms—such as real-time calibration routines that adjust for detected delays during an experiment—could further enhance the platform’s precision. Such algorithms might monitor timing discrepancies dynamically and adjust subsequent trial timings to compensate for systematic delays, thereby ensuring more consistent temporal measurements across varying devices and network conditions.

## Data Availability

The raw data supporting the conclusions of this article will be made available by the authors, without undue reservation.

## Footnotes

1
https://www.mpi.nl/idlas-nl

2 In Hintz et al. (2025), we noted that the lack of measurement invariance was more pronounced for the auditory than for the visual processing speed construct. We noted that this effect might be rooted in the fact that auditory stimulus presentation is more prone to imprecision than visual stimulus presentation.
