# Supplementary material for: Timing precision of the Individual Differences in Dutch Language Skills (IDLaS-NL) test battery
Source: Front Hum Neurosci. 2025 Sep 17;19:1625756. doi: 10.3389/fnhum.2025.1625756 (PMC12486307; doi:10.3389/fnhum.2025.1625756)
Supplement: Supplementary file 1 [file Image_1.pdf]

Supplementary materials 1: WEB EXPERIMENT ANALYZER—Module overview

Module – Arduino Uno (no image provided due to copyright)
Purpose: Main controller and connected to PC for data transfer (USB).

Module – VURectifier (page 2)
Purpose: The VURectifier converts line-in level analog audio signal (AC) via a double rectifier into voltage (DC). This DC signal is sampled by the Arduino Uno.
Interface: The analog input on the Arduino Uno.

Module – VUBar module V2 (page 3)
Purpose: The Arduino Uno roads the audio signal as a bar graph made of LEDs and lights a trigger LED when the dB-level reaches -21dB. There are a total of 3 buttons to adjust the trigger sensitivity: Decrement, Auto, and Increment. For the best results, the input signal on the VURectifier can be adjusted so that all segments on the bar graph are on.
Interface: SPI + I/O pin on the Arduino Uno.

Module – DTMF (page 4)
Purpose: An onboard DTMF module (from AliExpress) is used for converting incoming DTMF audio tones (signals) into corresponding digital numbers.

Module – DTMF Shield (page 5)
Purpose: An onboard DTMF module (from AliExpress) is used for converting incoming DTMF audio tones (signals) into corresponding digital numbers. The system also includes 3 switches, arranged in a matrix for PC keyboard control. A 1kHz test tone is generated by an ATtiny85 microcontroller, which is controlled by the Arduino Uno. If connected, the decoded DTMF output can optionally be displayed on a 7-segment display.
Interface: SPI + I/O pin on the Arduino Uno.

Module – Connector shield (page 6)
Purpose: The Arduino Uno is interconnected with the described modules to coordinate in- and output. One output pin controls a PC keyboard matrix by using an optocoupler TLP175A, which, effectively emulates a button (spacebar) press.
Interface: SPI + I/O pin on the Arduino Uno.

Module – Big 7Segment Display (page 7)
Purpose: Displays the DTMF code when an audio tone is present at the DTMF input (handled via software). The original idea was to record the experiment by means of a high-speed video camera and use the displayed code to track the current trial. However, this feature was never used.
Interface: SPI + I/O pin on the Arduino Uno.

Module – USB Keyboard logic board salvaged from a PC keyboard (no image available)
Purpose: To generate (emulate) a spacebar press and release via software, connect the board to a PC and open a text editor like Notepad. Then, short the relevant row and column pins on the keyboard matrix. This will simulate a spacebar press, which should provide an output in Notepad.
Interface: SPI + I/O pin on the Arduino Uno.

Module – LIGHT-TO-VOLTAGE OPTICAL SENSOR – TSL250* (page 8)
Purpose: Positioned directly over a region of a TFT screen to detect black/white visual markers used to measure timing precision.
Interface: Analog out connected to Arduino UNO (Opto 1 or Opto 2) input.

Module – VURectifier
Purpose: The VURectifier converts line-in level analog audio signal (AC) via a double rectifier into voltage (DC). This DC signal is sampled by the Arduino Uno.
Interface: The analog input on the Arduino Uno.

2

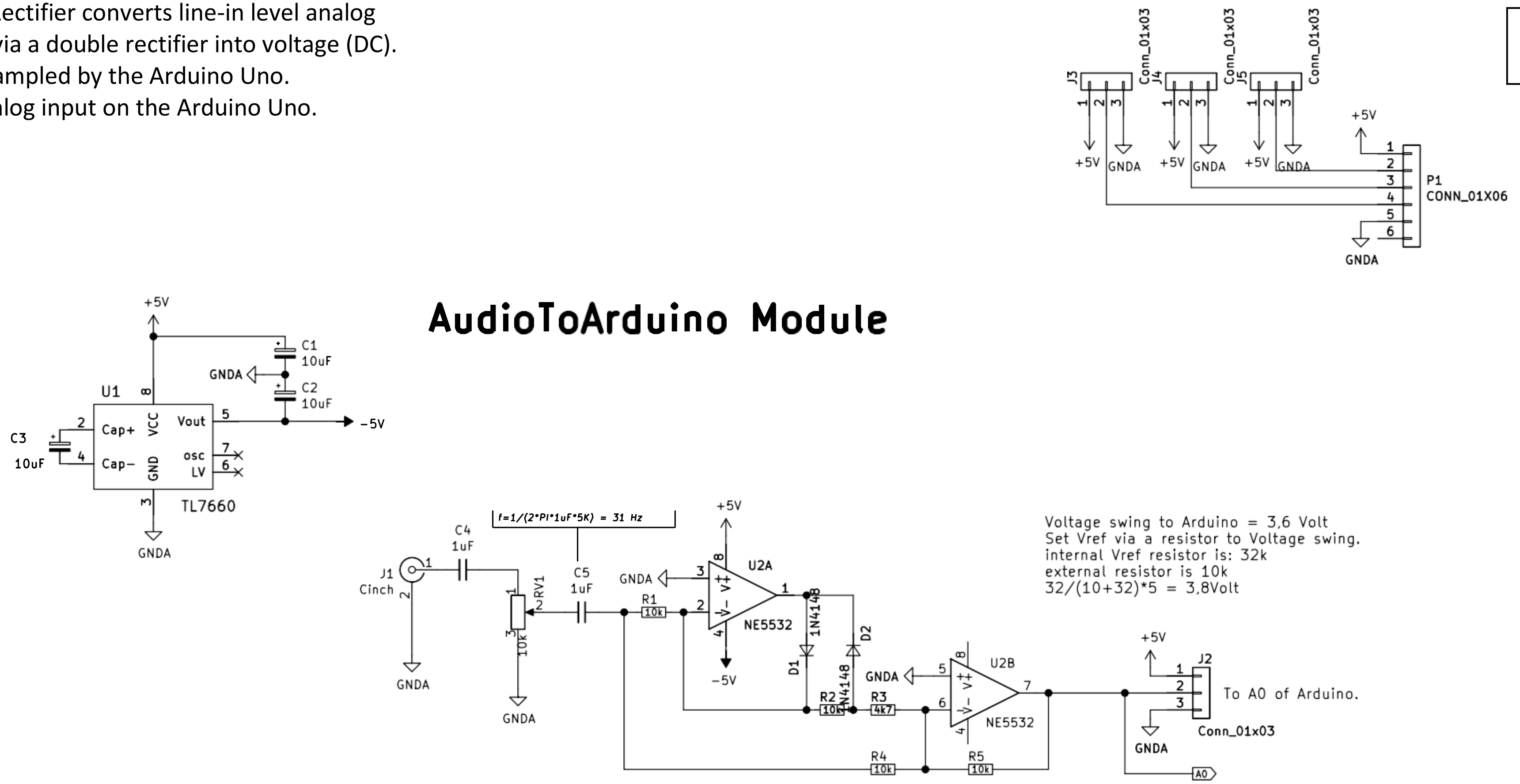

M1 3,5mm
M2 3,5mm
M3 3,5mm
M4 3,5mm

Max Planck Institute

Designer: JAH Weustink
Adres: Wundtlaan 1
Postcode: 6525XD
Plaats: Nijmegen

Sheet: /
File: VUrectifier.kicad_sch

Title: VUrectifier module

Size: A4 | Date: 26-10-2017 | **Rev: 0**
KiCad E.D.A. 8.0.8 | Id: 1/1

Module – VUBar module V2
Purpose: The Arduino Uno roads the audio signal as a bar graph made of LEDs and lights a trigger LED when the dB-level reaches -21dB. There are a total of 3 buttons to adjust the trigger sensitivity: Decrement, Auto, and Increment. For the best results, the input signal on the VURectifier can be adjusted so that all segments on the bar graph are on.
Interface: SPI + I/O pin on the Arduino Uno.

3

VUbar Arduino Module

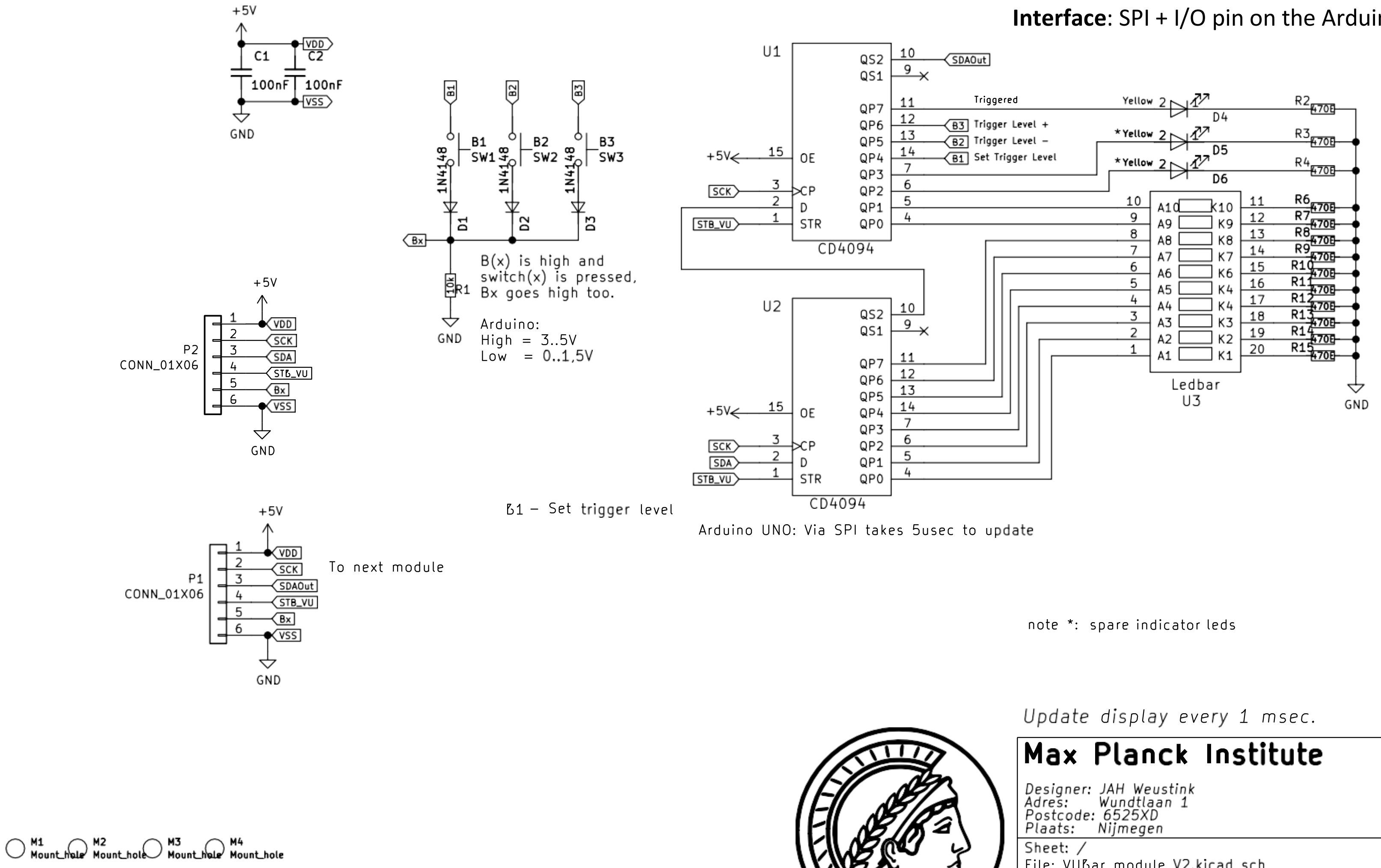

Max Planck Institute

Designer: JAH Weustink
Adres: Wundtlaan 1
Postcode: 6525XD
Plaats: Nijmegen

Sheet: /
File: VUBar module V2.kicad_sch

Title: VUbar module

Size: A4	Date: 26-10-2017	**Rev: 0**
KiCad E.D.A. 8.0.8		Id: 1/1

Module – DTMF

Purpose: An onboard DTMF module (from AliExpress) is used for converting incoming DTMF audio tones (signals) into corresponding digital numbers.

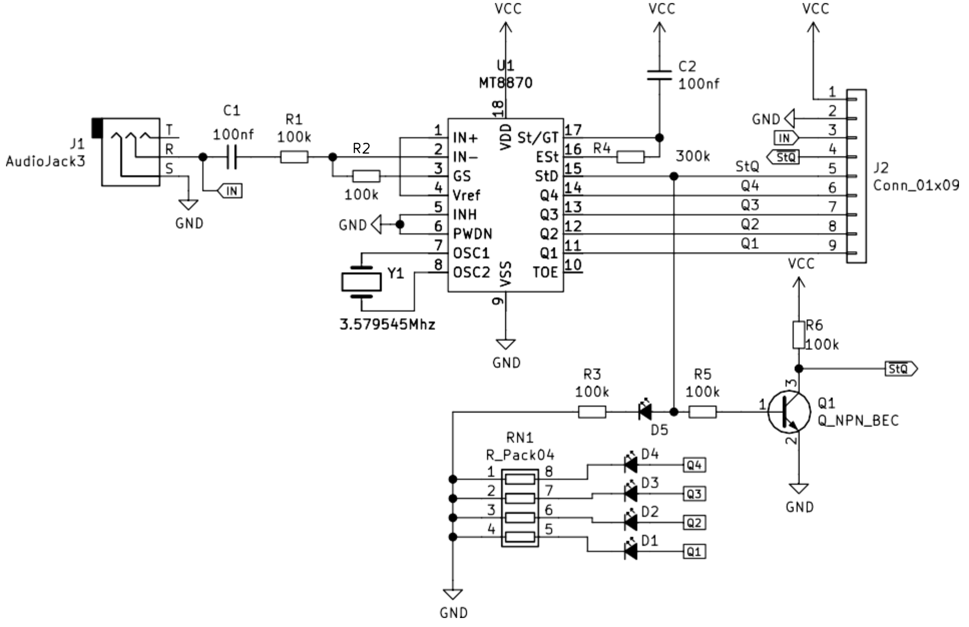

Ordered from: TinyTronics, MT8870 DTMF ModuleMT8870 DTMF Module

Max Planck Institute

Designer: JAH Weustink
Adres: Wundtlaan 1
Postcode: 6525XD
Plaats: Nijmegen

Sheet: /
File: DTMF_module.kicad_sch

Title: DTMF module

Size: A4	Date: 30-11-2020	**Rev:**
KiCad E.D.A. 8.0.8		Id: 1/1

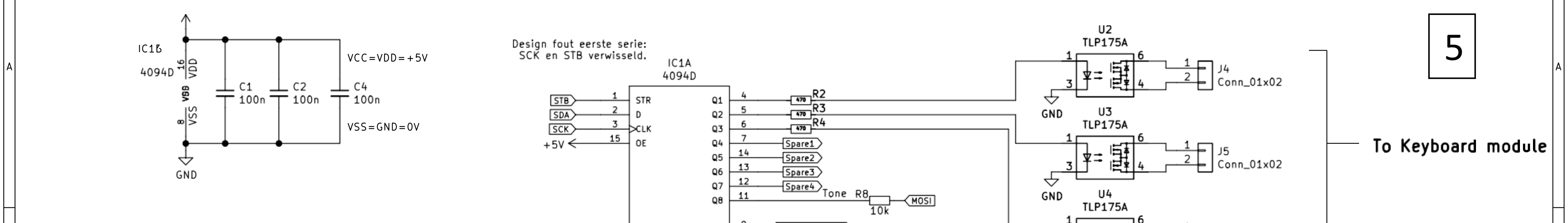

Module – DTMF Shield
Purpose: An onboard DTMF module (from AliExpress) is used for converting incoming DTMF audio tones (signals) into corresponding digital numbers. The system also includes 3 switches, arranged in a matrix for PC keyboard control. A 1kHz test tone is generated by an ATtiny85 microcontroller, which is controlled by the Arduino Uno. If connected, the decoded DTMF output can optionally be displayed on a 7-segment display.
Interface: SPI + I/O pin on the Arduino Uno.

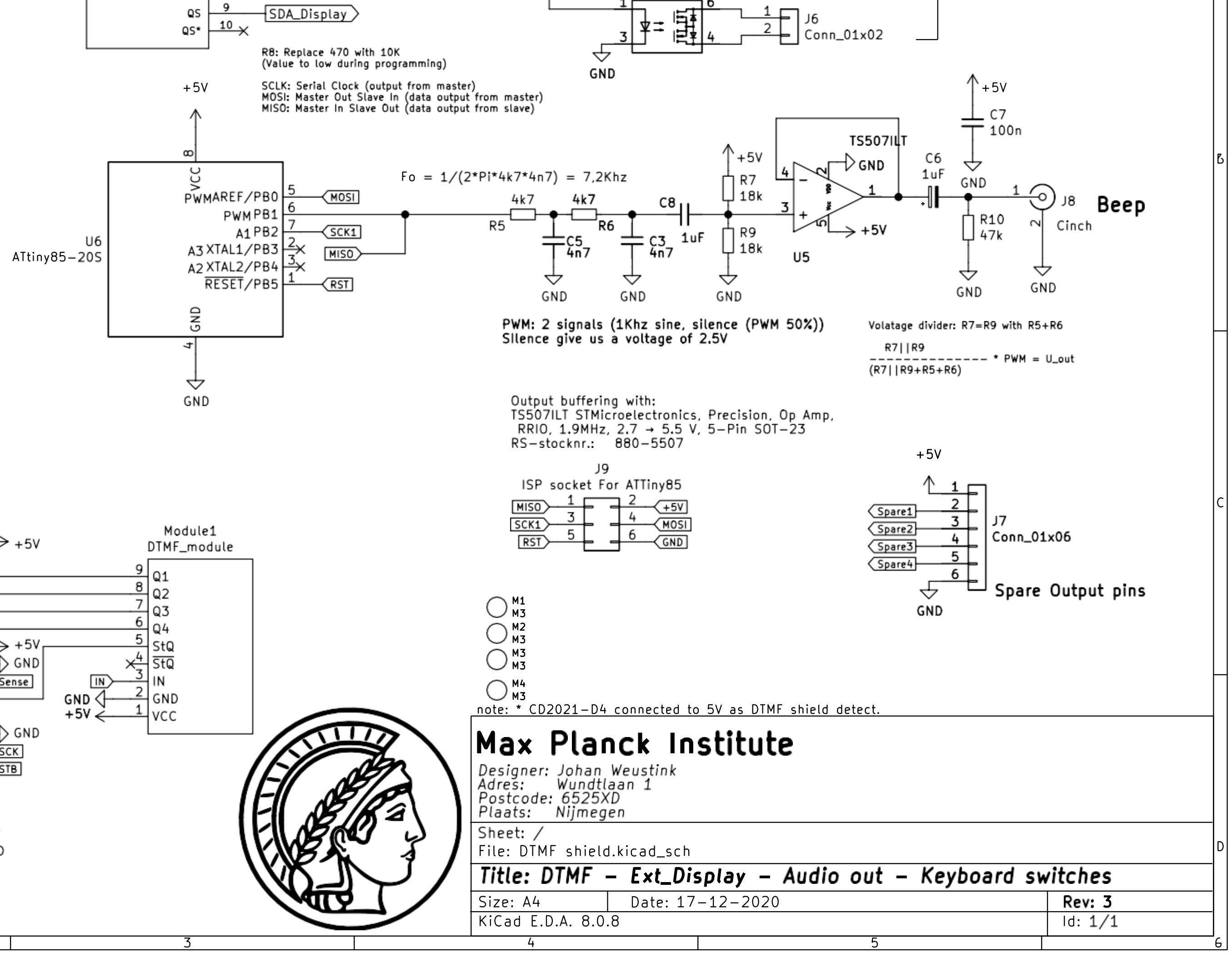

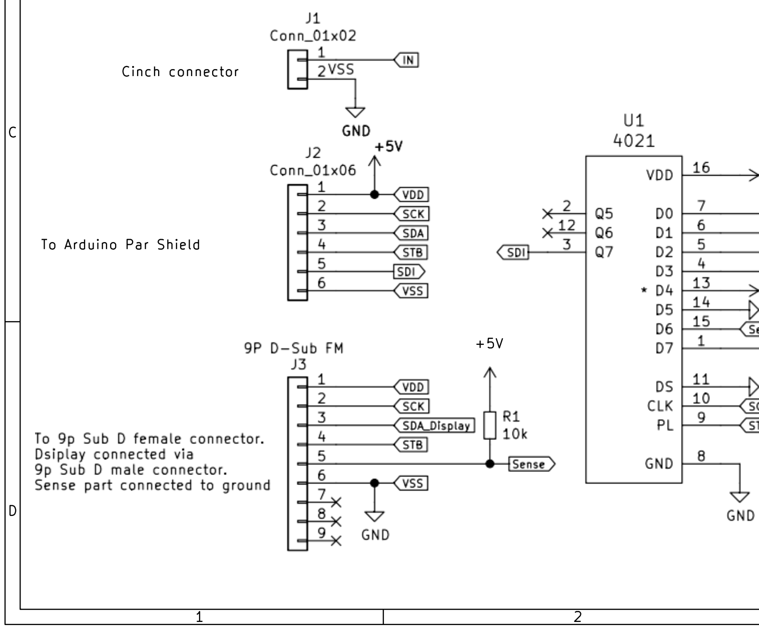

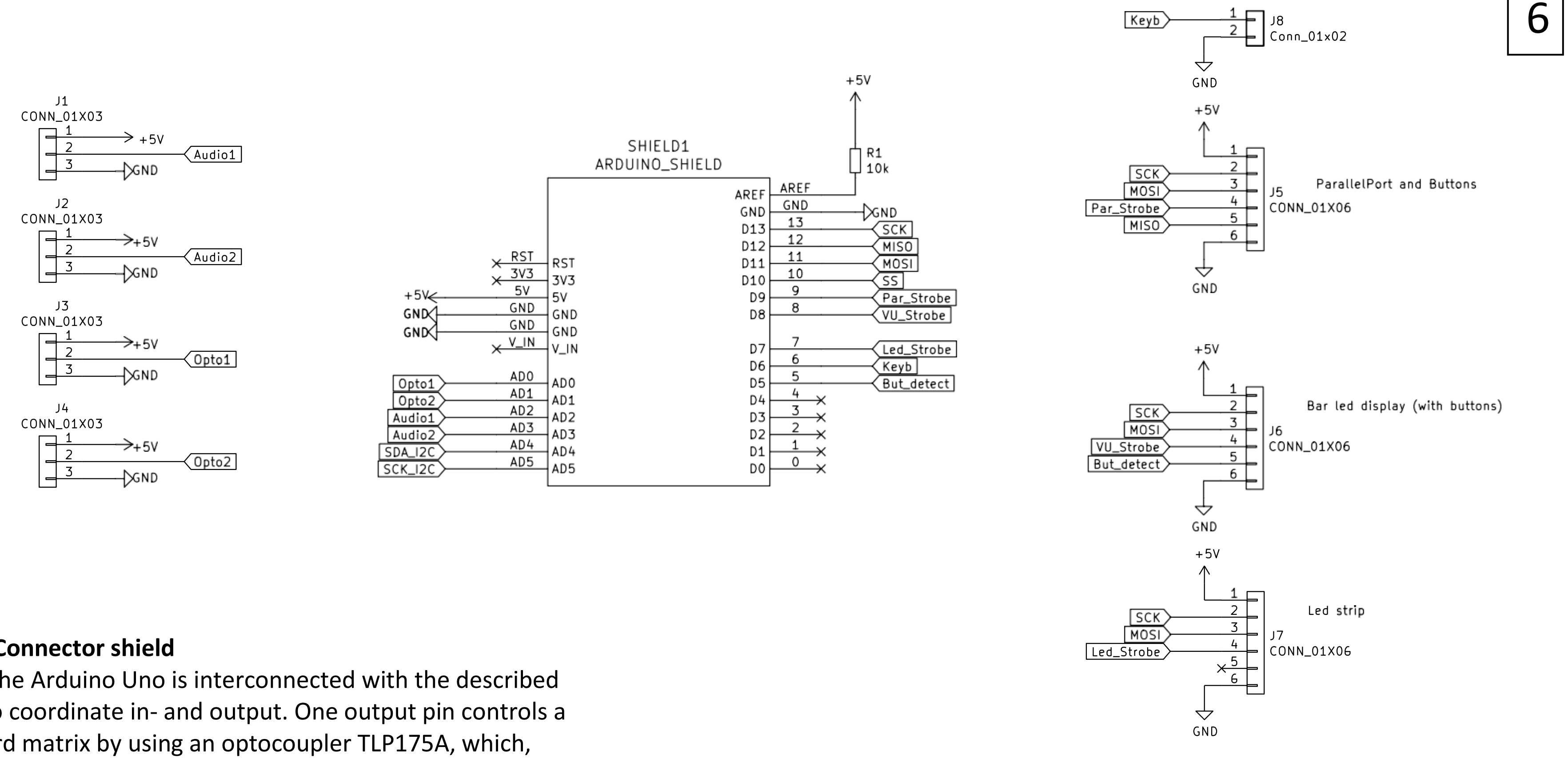

Module – Connector shield

Purpose: The Arduino Uno is interconnected with the described modules to coordinate in- and output. One output pin controls a PC keyboard matrix by using an optocoupler TLP175A, which, effectively emulates a button (spacebar) press.

Interface: SPI + I/O pin on the Arduino Uno.

Max Planck Institute

Designer: JAH. Weustink
Adres: Wundtlaan 1
Postcode: 6525XD
Plaats: Nijmegen

Sheet: /
File: Connector shield.kicad_sch

Title: Connector schield

Size: A4 | Date: 25-8-2020 | **Rev:**
KiCad E.D.A. 8.0.8 | Id: 1/1

Module – Big 7Segment Display
Purpose: Displays the DTMF code when an audio tone is present at the DTMF input (handled via software). The original idea was to record the experiment by means of a high-speed video camera and use the displayed code to track the current trial. However, this feature was never used.
Interface: SPI + I/O pin on the Arduino Uno.

7

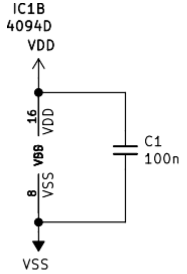

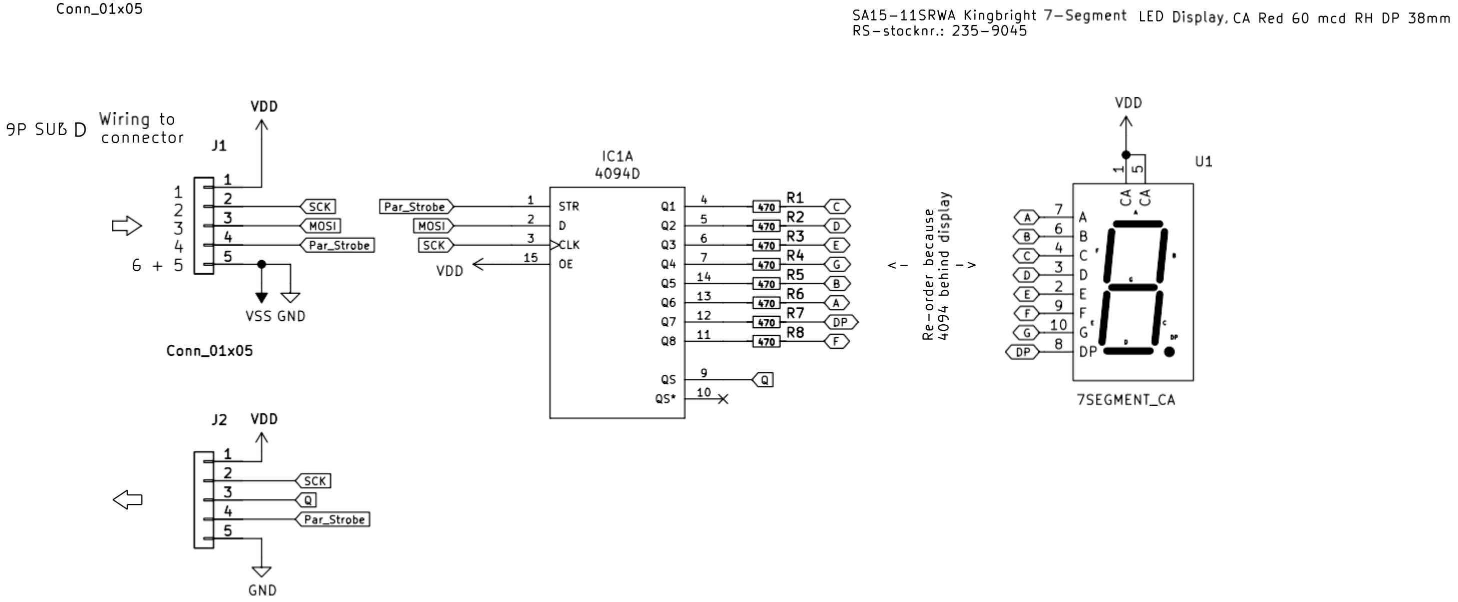

Max Planck Institute
Designer: JAH Weustink
Adres: Wundtlaan 1
Postcode: 6525XD
Plaats: Nijmegen
Sheet: /
File: Big 7Segment Display.kicad_sch
Title: Big 7segment display
Size: A4 Date: 25-8-2021 **Rev:**
KiCad E.D.A. 8.0.8 Id: 1/1

Module – LIGHT-TO-VOLTAGE OPTICAL SENSOR – TSL250*
Purpose: Positioned directly over a region of a TFT screen to detect black/white visual markers used to measure timing precision.
Interface: Analog out connected to Arduino UNO (Opto 1 or Opto 2) input.
*This product is no longer manufactured.

8

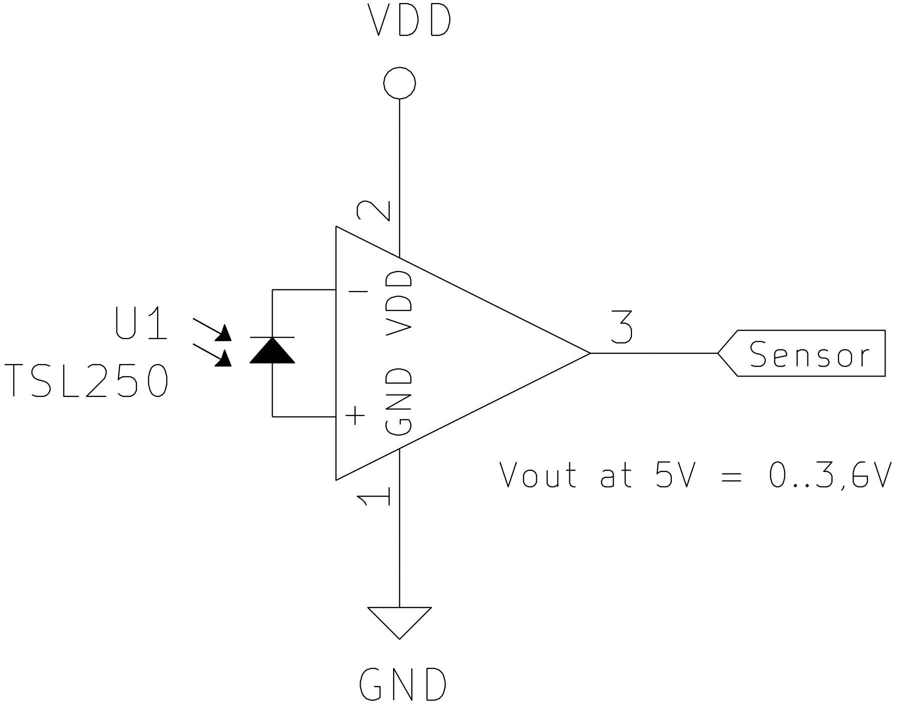

**Max Planck Institute**		
Designer: JAH Weustink		
Adres: Wundtlaan 1		
Postcode: 6525XD		
Plaats: Nijmegen		
Sheet: /		
File: OptoSensor.kicad_sch		
Title: Simple Opto Sensor		
Size: A4	Date: 2-1-2018	**Rev: 0**
KiCad E.D.A. 8.0.8		Id: 1/1
