# Supplementary material for: Timing precision of the Individual Differences in Dutch Language Skills (IDLaS-NL) test battery
Source: Front Hum Neurosci. 2025 Sep 17;19:1625756. doi: 10.3389/fnhum.2025.1625756 (PMC12486307; doi:10.3389/fnhum.2025.1625756)

Supplementary materials 3: timing precision for each measure in each of the five tests in study 2

Figure 1

Timing of trial events from fixation cross onset to button response (upper panel) and cumulative frequency of timing imprecision (bottom panel) for the Auditory simple reaction time test. Each plot represents a precision event, with a baseline and the corresponding timing deviations as recorded by the Mac and Windows devices.

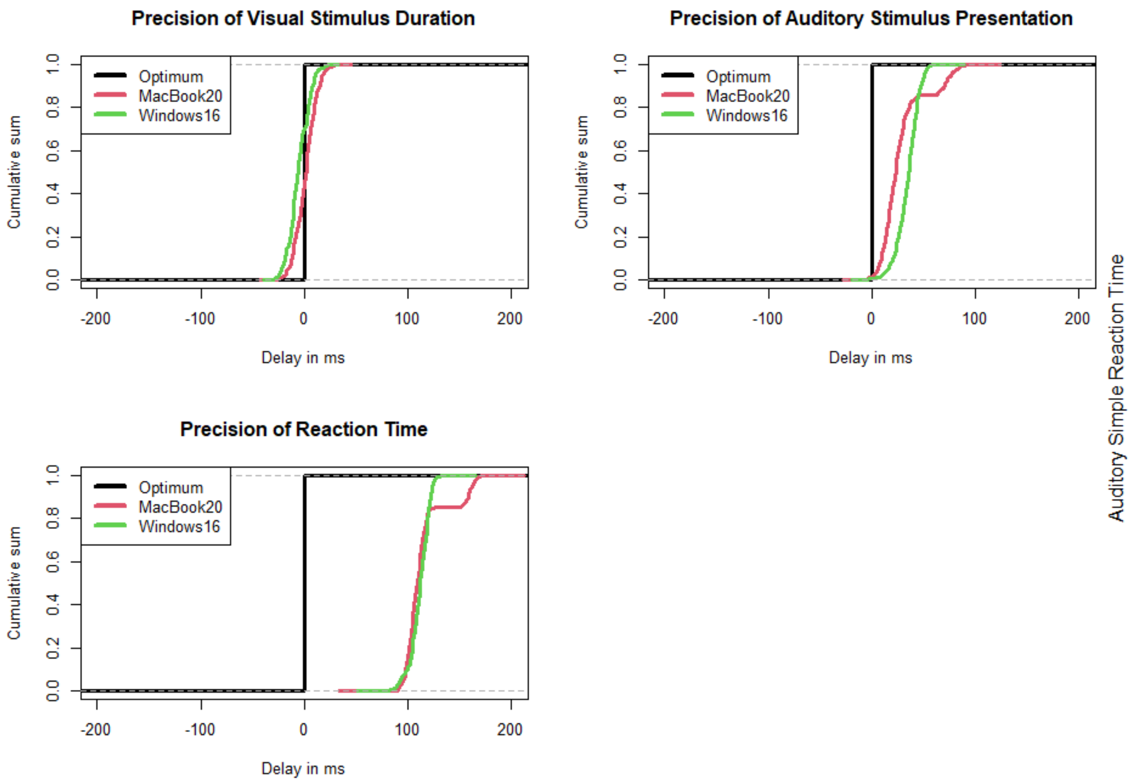

Figure 2

Timing of trial events from fixation cross onset to button response (upper panel) and cumulative frequency of timing imprecision (bottom panel) for the Visual simple reaction time test. Each plot represents a precision event, with a baseline and the corresponding timing deviations as recorded by the Mac and Windows devices.

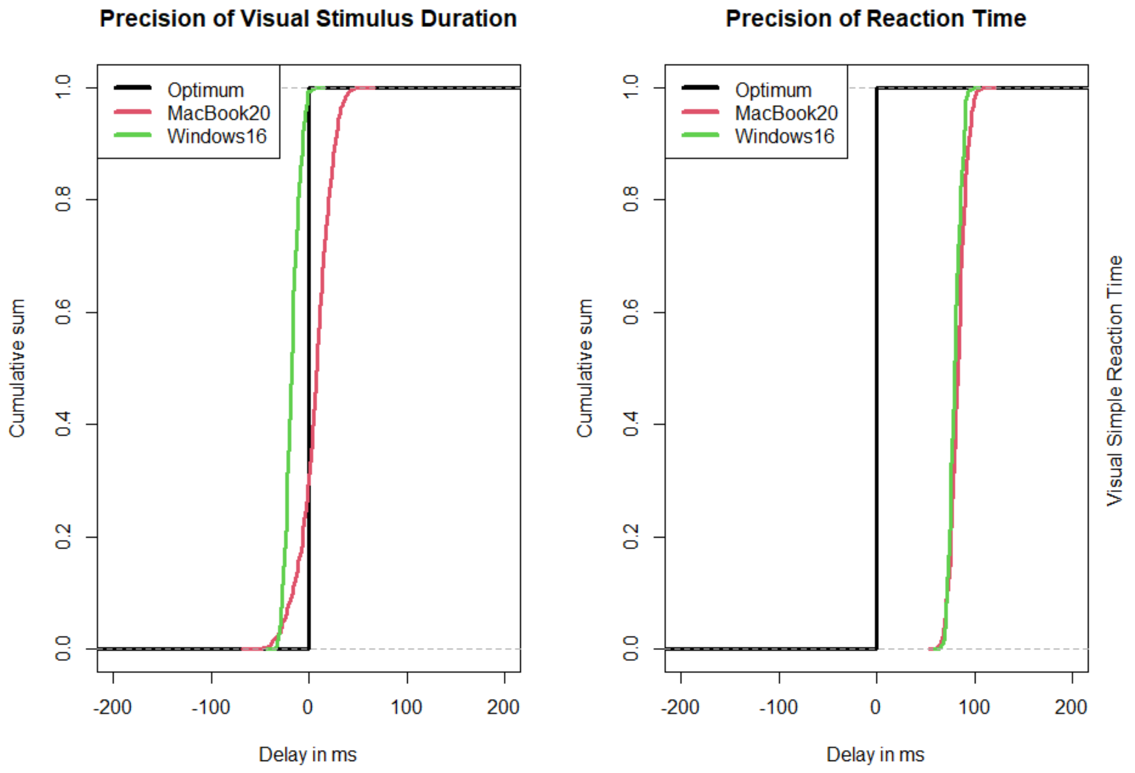

Figure 3

Timing of trial events from fixation cross onset to button response (upper panel) and cumulative frequency of timing imprecision (bottom panel) for the Picture naming test. Each plot represents a precision event, with a baseline and the corresponding timing deviations as recorded by the Mac and Windows devices.

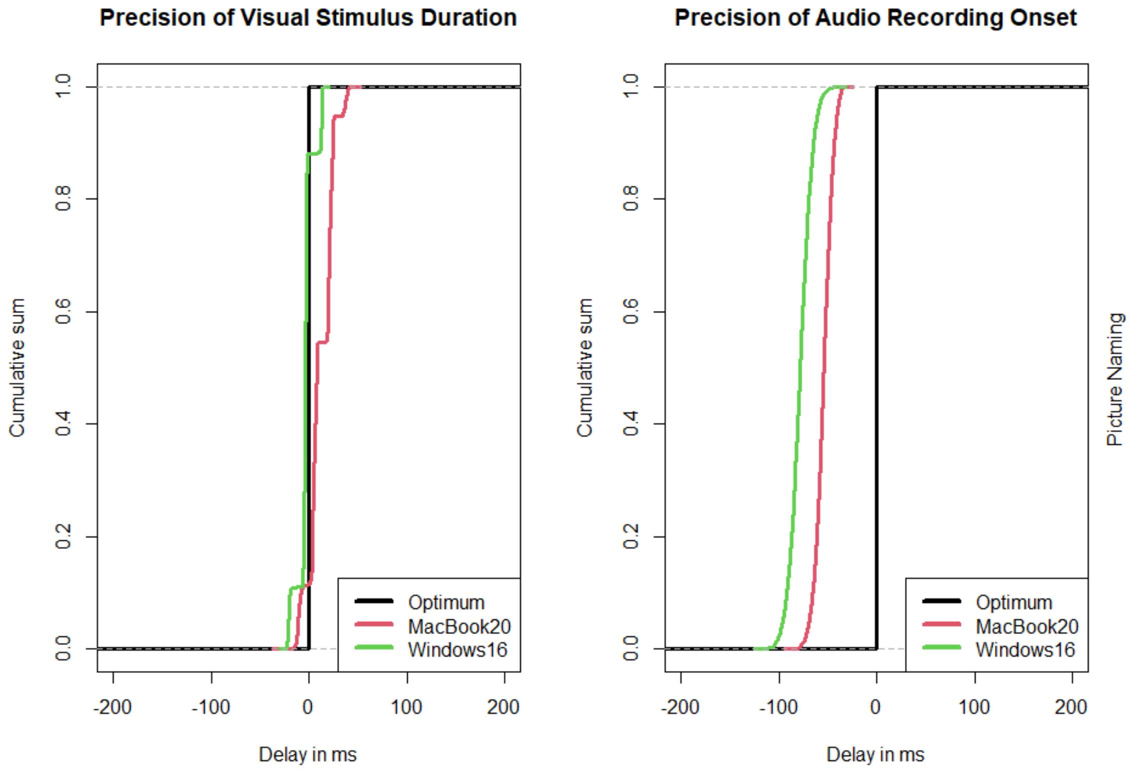

Figure 4

Timing of trial events from fixation cross onset to button response (upper panel) and cumulative frequency of timing imprecision (bottom panel) for the Structured sentence generation test. Each plot represents a precision event, with a baseline and the corresponding timing deviations as recorded by the Mac and Windows devices.

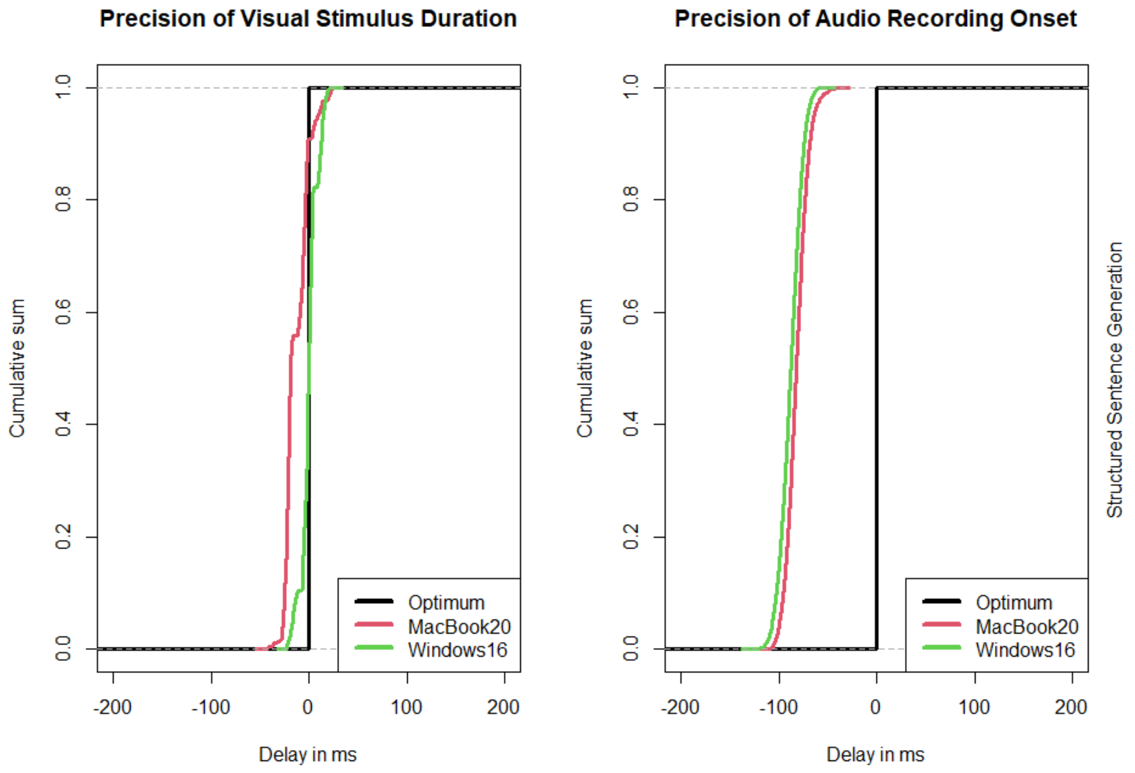

Figure 5

Timing of trial events from fixation cross onset to button response (upper panel) and cumulative frequency of timing imprecision (bottom panel) for the Sentence comprehension test. Each plot represents a precision event, with a baseline and the corresponding timing deviations as recorded by the Mac and Windows devices.

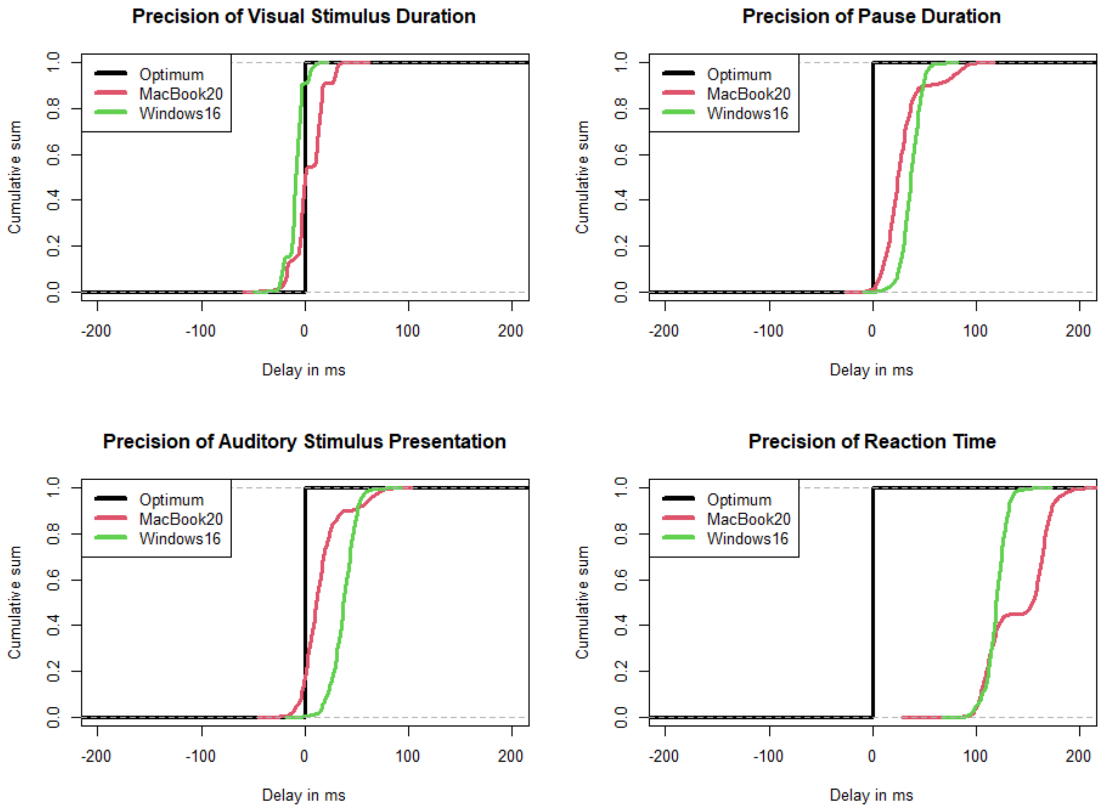

Supplement: Supplementary file 3 [file Image_3.pdf]
